# Fulminant Course of Neuromyelitis Optica in a Patient With Anti-MDA5 Antibody-Positive Dermatomyositis: A Case Report

**DOI:** 10.3389/fmed.2020.576436

**Published:** 2020-11-11

**Authors:** You-Ri Kang, Kun-Hee Kim, Tai-Seung Nam, Kyung-Hwa Lee, Kyung Wook Kang, Seung-Jin Lee, Seok-Yong Choi, Gopalakrishnan Chandrasekaran, Myeong-Kyu Kim

**Affiliations:** ^1^Department of Neurology, Chonnam National University Medical School, Chonnam National University Hospital, Gwangju, South Korea; ^2^Department of Biomedical Sciences, Chonnam National University Medical School, Gwangju, South Korea; ^3^Department of Pathology, Chonnam National University Medical School, Gwangju, South Korea; ^4^Department of Radiology, Chonnam National University Medical School, Gwangju, South Korea

**Keywords:** neuromyelitis optica, clinically amyopathic dermatomyositis, interstitial lung disease, antibody, rituximab, ferritin

## Abstract

Anti-melanoma differentiation-associated gene 5 (anti-MDA5) antibody is a myositis-specific marker detected in clinically amyopathic dermatomyositis (DM). DM with anti-MDA5 antibody can be accompanied by rapidly progressive interstitial lung disease (RP-ILD) and other autoimmune disorders. Until now, only one case of neuromyelitis optica (NMO) with anti-MDA5-positive DM has been reported worldwide, in which the patient achieved a favorable outcome with intensive immunotherapy. We report a case of NMO in a patient with anti-MDA5-positive DM complicated by ILD and rheumatoid arthritis. Our patient experienced a fulminant course of NMO, rather than RP-ILD, in the presence of hyperferritinemia, which resulted in profound neurological sequelae despite immunotherapy including rituximab.

## Introduction

Anti-melanoma differentiation-associated gene 5 (anti-MDA5) antibody is a myositis-specific marker detected in clinically amyopathic dermatomyositis (DM) (1). Anti-MDA5-positive DM is known to be complicated by rapidly progressive interstitial lung disease (RP-ILD), resulting in high mortality (2, 3). Serum ferritin level is a disease activity biomarker in interstitial lung disease (ILD) with anti-MDA5-positive DM (4). Neuromyelitis optica (NMO) is an immune-mediated inflammatory disease of the central nervous system whose pathogenesis is linked to anti-aquaporin-4 (anti-AQP4) antibody (5). To the best of our knowledge, only one case of NMO with anti-MDA5-positive DM has been reported worldwide, in which the patient achieved a favorable outcome with intensive immunotherapy (6). Herein, we report a case of NMO with high disease activity in the presence of anti-MDA5 antibody and persistent hyperferritinemia, which resulted in profound neurological sequelae.

## Case Presentation

A 35-year-old man who had a 2-month history of urinary difficulty was referred to our neurology clinic for evaluation of decreased visual acuity of the right eye and paresthesia of both legs. Within the last 2 months, he had been diagnosed with rheumatoid arthritis (RA), ILD, and clinically amyopathic DM.

**Figure 1:**
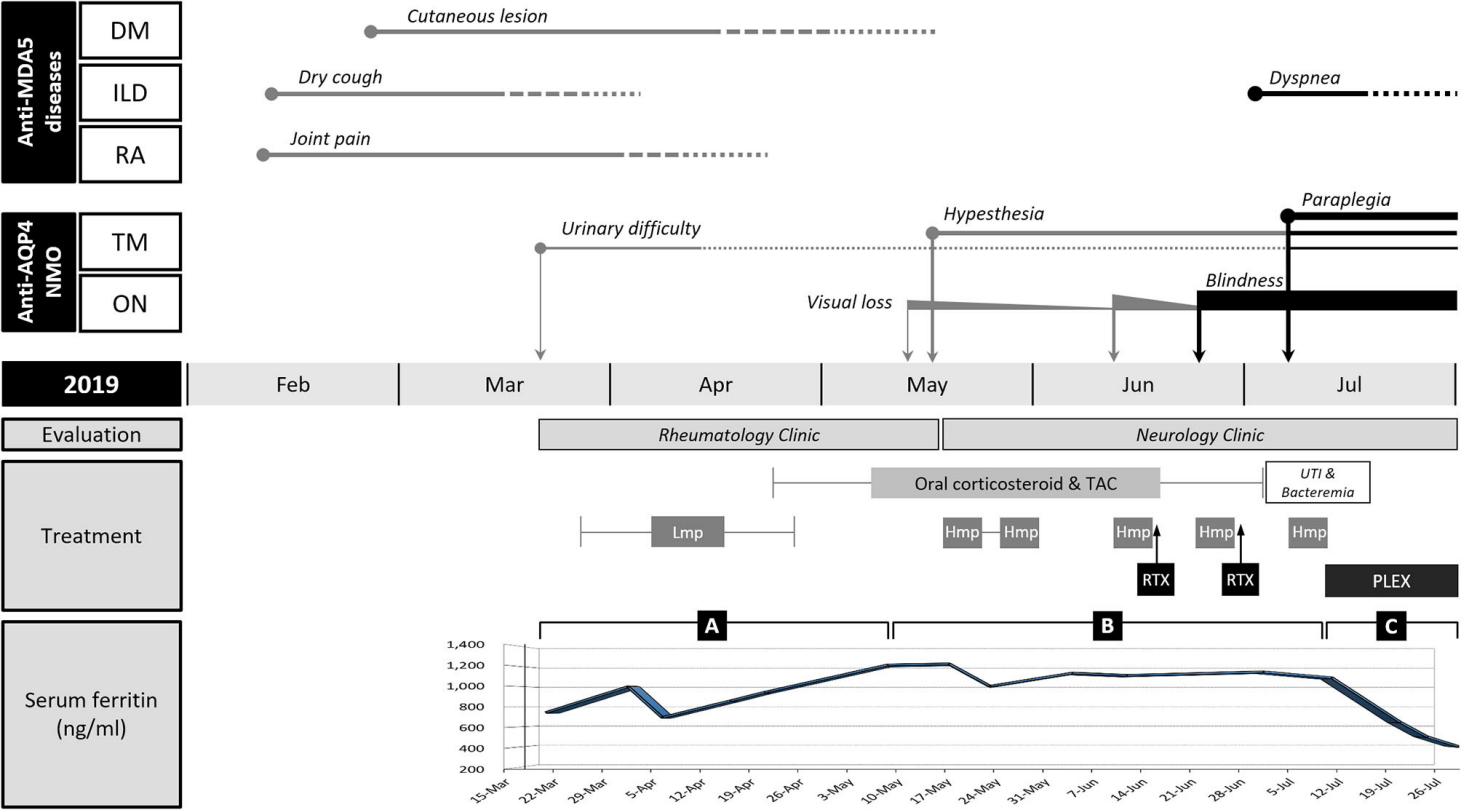
Clinical course and changes in the serum ferritin level of the patient. Anti-MDA5-positive dermatomyositis (DM), interstitial lung disease (ILD), and rheumatoid arthritis (RA) were improved after immunotherapy with corticosteroids and tacrolimus (TAC), but neuromyelitis optica (NMO) frequently relapsed from mid-May (in 2019). NMO and ILD were exacerbated after rituximab (RTX) induction. Hyperferritinemia persisted throughout the course of illness. The change of serum ferritin was divided into three segments based on the inflection points: rising **(A)**, plateau **(B)**, and falling **(C)** phases. **(A)** Serum ferritin level initially decreased by 30% at the beginning of immunotherapy but kept increasing despite symptomatic improvement of ILD. **(B)** Serum ferritin level remained high in the period of frequent relapses of NMO. **(C)** Serum ferritin level drastically dropped after plasma exchange (PLEX). The length of the straight line indicates the duration of symptoms or signs. The thickness of straight lines and the size of circles or rectangles directly indicate clinical severity. The solid line and dotted lines reflect the presence and alleviation of the symptoms, respectively. TM, transverse myelitis; ON, optic neuritis; Lmp, intravenous low-dose methylprednisolone; Hmp, intravenous high-dose methylprednisolone; UTI, urinary tract infection.

**Figure 2:**
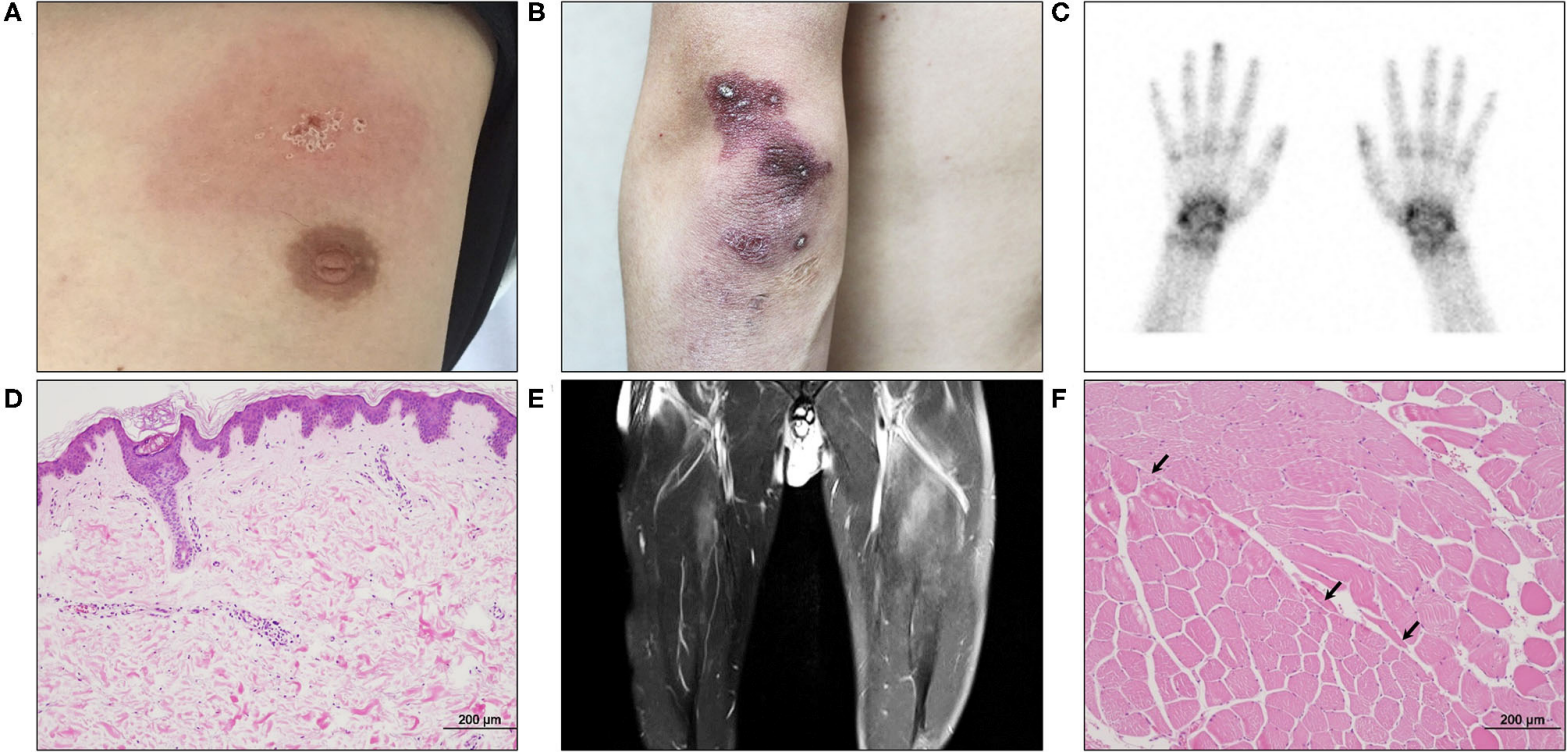
The initial cutaneous, imaging, and pathological findings of the patient. **(A)** An erythematous patch with scales was seen in the anterior chest. **(B)** A diffuse purpuric patch with crusts was seen in the left elbow. **(C)** The three-phase bone scintigraphy revealed increased uptake in both wrists and interphalangeal joints. **(D)** Skin biopsy of the chest displayed a couple of small vessels with perivascular inflammatory cell infiltration in the upper dermis (H&E, ×100). **(E)** Thigh MRI showed multifocal infiltrations in the anterior thigh muscles with left side dominancy. **(F)** Biopsy of the left vastus lateralis muscle revealed a small number of atrophic angular myocytes (arrows) in the perifascicular area (H&E, ×100).

When the patient first visited the rheumatology clinic, he had a 4-week history of non-productive cough and joint pain with morning stiffness in both hands. The detailed clinical course of the patient is shown in Figure 1. On physical examination, erythematous or purpuric patches with crusts on the anterior chest, knuckles, and elbows (Figures 2A,B) were observed. Ultrasonography of joints showed synovitis and effusion involving multiple small joints (both wrist joints and five small joints of both hands) and both knee joints. The three-phase bone scintigraphy revealed inflammation in the affected joints (Figure 2C). Computed tomography (CT) of the chest showed perilobular consolidations and fibrosis in the posterior aspect of both lower lungs (**Figure 4A**). In pulmonary function test (PFT), forced vital capacity (FVC) was decreased (3.05 L, 61% of predicted value), and forced expiratory volume in 1 s (FEV1)/FVC ratio was increased (93%), which were suggestive of restrictive lung disease. The serologic test showed positivity for antinuclear antibody (titer 1:40), rheumatoid factor (59.4 IU/ml, normal range <14 IU/ml), and anti-cyclic citrullinated peptide antibody (9.5 U/ml, normal range <5 U/ml). The levels of acute phase reactants including erythrocyte sedimentation rate (ESR), C-reactive protein (CRP), and serum ferritin were elevated as follows: ESR 66 mm/h (normal range <20 mm/h), CRP 1.03 mg/dl (normal range <0.3 mg/dl), and ferritin 743 ng/ml (normal range 4–275 ng/ml). Skin biopsy of the anterior chest showed superficial perivascular inflammation (Figure 2D).

Under the diagnosis of RA-ILD, the patient started intravenous (IV) methylprednisolone therapy. A week after hospitalization, a sudden elevation (2,187 IU/L, normal range 56–244 IU/L) of serum creatine kinase (CK) was observed. Though the patient did not complain of muscle weakness and myalgia, magnetic resonance imaging (MRI) of the thigh showed contrast-enhancing asymmetric and patchy T2-hyperintensity in hip and thigh muscles (Figure 2E). Anti-synthetase antibodies and anti-Mi-2 antibodies were negative. The pathologic finding of muscle biopsy led to additional diagnosis of DM (Figure 2F). The patient continued IV methylprednisolone therapy (20–40 mg daily) for a month and took oral deflazacort (24 mg daily) and tacrolimus (1–2 mg daily) for the next month. The symptoms and signs including arthralgia and cough almost improved, and the levels of muscle enzymes normalized. Nevertheless, serum ferritin level has been gradually increasing up to 1,210 ng/ml after a transient decrease at the beginning of immunotherapy (*Segment A* in Figure 1).

**Figure 3:**
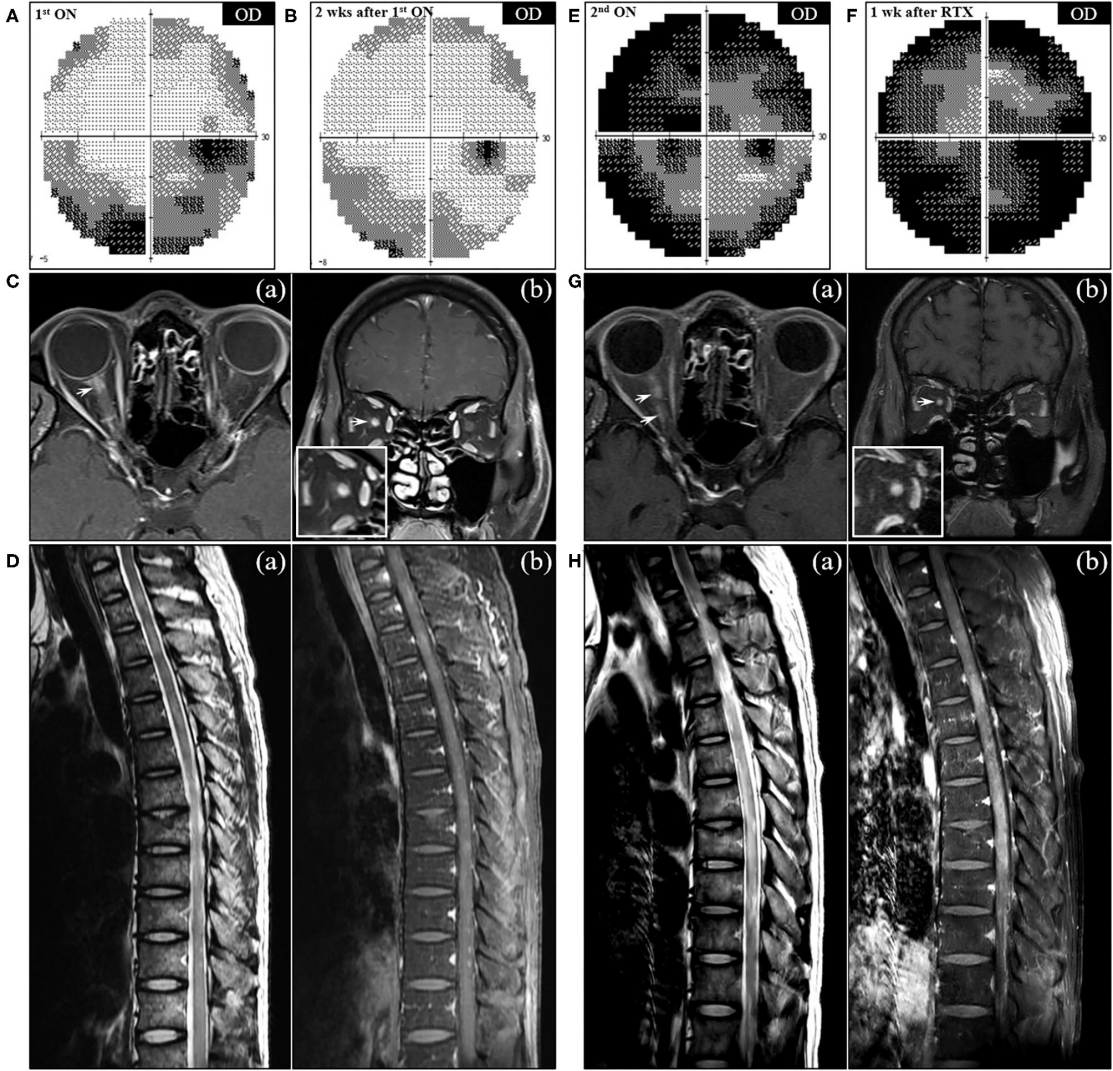
Serial visual field tests and MRI findings of NMO in the patient. **(A)** The initial visual field (VF) test demonstrated the incomplete loss of the inferior and peripheral vision in the right eye, which partially improved after corticosteroid therapy **(B)**. **(C)** Axial (a) and coronal (b) T1-weighted orbit MR images showed contrast enhancement in the right optic nerve at the back of the eye (arrow). **(D)** Sagittal MRI of the thoracic spine showed multifocal T2-hyperintensity (a) with patchy contrast enhancement (b). **(E)** VF test performed at the second attack of ON showed near-total blindness of the right eye, which was exacerbated to complete blindness a week after rituximab injection **(F)**. **(G)** Follow-up orbit MRI showed the contrast-enhancing lesions (arrows) affecting the entire right optic nerve on the axial (a) and coronal (b) T1-weighted images. **(H)** Follow-up spine MRI after the second rituximab injection showed long segmental T2-hyperintensity (a) with diffuse enhancement (b) in the thoracic cord extending from T4 to T11 vertebral levels. OD, oculus dexter.

**Figure 4:**
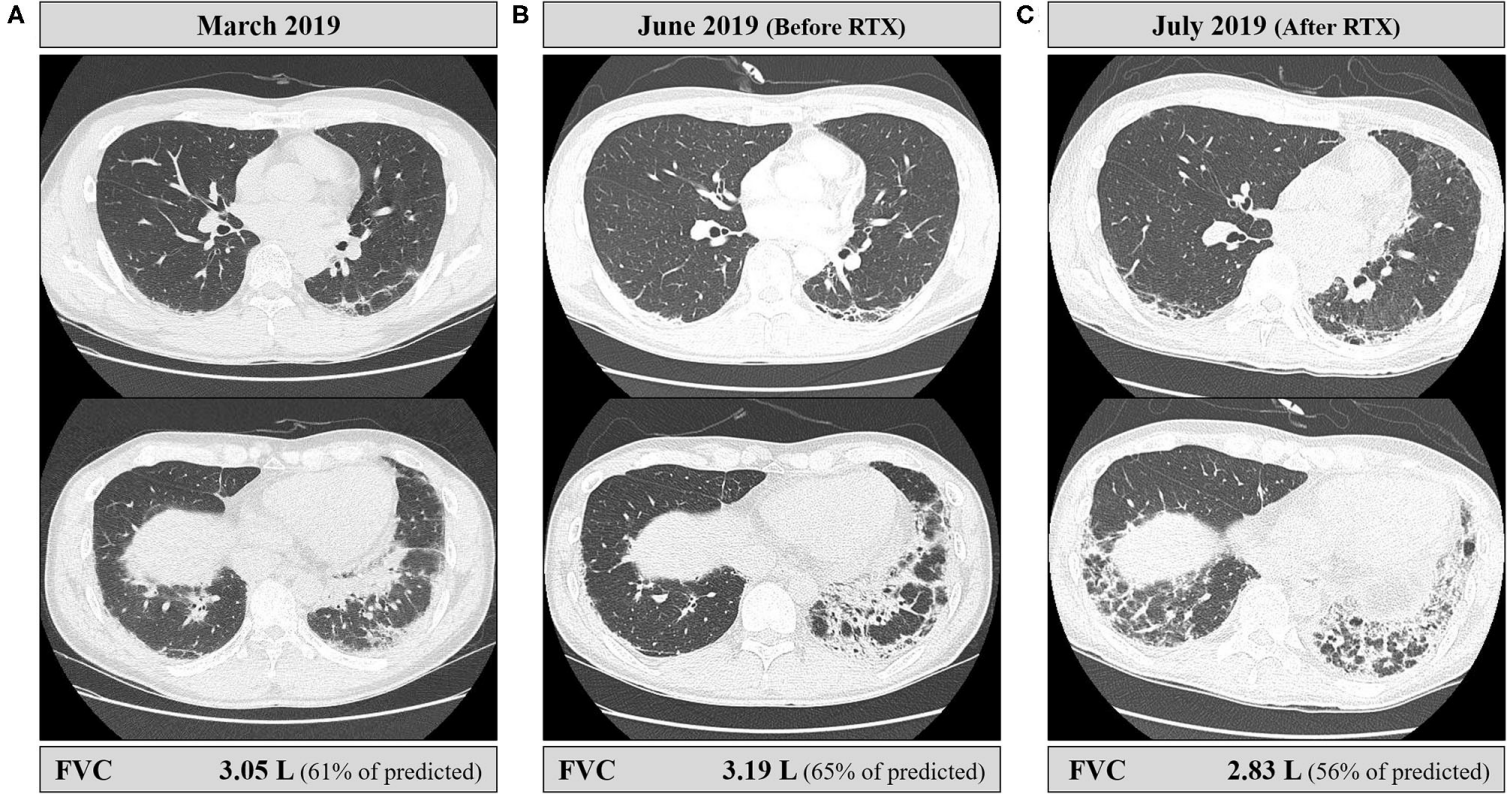
Serial chest CT findings and pulmonary functions of the patient. **(A)** Initial CT at the time of diagnosis of ILD showed perilobular consolidations and fibrosis in posterior aspects of the lower lobes, and forced vital capacity (FVC) was 3.05 L. **(B)** CT performed just before the rituximab injection showed the increased extent of fibrosis. However, the patient did not complain of respiratory symptoms including cough or dyspnea, and FVC was slightly improved by 4.6% compared to baseline. **(C)** CT performed immediately after the second rituximab injection showed an increased extent of fibrosis and perilobular consolidations. The patient developed mild dyspnea, and FVC declined by 11% from the latest one.

When the patient presented to a neurology clinic with visual and sensory symptoms, it had been 3 months since the onset of symptoms of RA and ILD. Neurological examination showed dilated pupil of the right eye with responsiveness, paresthesia below T4 dermatome, and bilaterally positive Babinski sign. Visual field (VF) test showed altitudinal hemianopia involving the lower hemisphere (Figure 3A). Brain MRI showed retrobulbar neuritis of the right optic nerve (Figure 3C) without abnormality in the brain. Spine MRI showed multifocal contrast-enhancing T2-hyperintensity involving the thoracic spinal cord (Figure 3D). CSF analysis showed elevated protein levels (72.8 mg/dl, normal range <45 mg/dl) without pleocytosis. Serologic study revealed positivity (1+) for anti-AQP4 antibody (measured by cell-based indirect immunofluorescence assay) and strong positivity (2,875 U/ml, normal value <32 U/ml) for anti-MDA5 antibody (measured by enzyme-linked immunosorbent assay). Ultimately, the patient was diagnosed with anti-AQP4-positive NMO combined with anti-MDA5-positive DM with ILD and RA. Two cycles of IV pulsed methylprednisolone (1,000 mg daily for five consecutive days) were injected a week apart, and the dose of tacrolimus was increased to 3 mg daily. The visual acuity got better gradually (Figure 3B), but sensory disturbance did not. Although pulmonary fibrosis seemed to be slightly progressed on the regular follow-up chest CT, his respiratory symptoms and FVC were rather improved (Figure 4B). Meanwhile, serum ferritin level constantly remained high at above 1,000 ng/ml (*Segment B* in Figure 1).

A month after the first attack of optic neuritis, his vision of the right eye suddenly worsened again. VF exam showed severe visual disturbance with periphery involvement (Figure 3E), and rituximab (IV, 1,000 mg each, 2 weeks apart) was decided to be administered to prevent further relapse of NMO. However, his VF defect was exacerbated after the first rituximab injection (Figure 3F), and orbit MRI demonstrated the relapse of optic neuritis (Figure 3G). Furthermore, the patient developed mild dyspnea (oxygen demand, 2 L/min via nasal prong) followed by complete paraplegia and lack of voiding after a subsequent rituximab injection. Spine MRI showed longitudinally extensive transverse myelitis involving the thoracic cord (Figure 3H). FVC declined to 2.83 L (56% of the predicted) in PFT, and ILD was aggravated on chest CT (Figure 4C). Fluorescence intensity of anti-AQP4 antibody significantly increased to 4+, even though CD19^+^ B-cells constituted 0.1% of the total lymphocytes. Oral immunosuppressants (prednisone 20 mg and tacrolimus 3 mg) were discontinued owing to recurrent urinary tract infection and subsequent *Klebsiella aerogenes* bacteremia, and the patient underwent eight sessions of therapeutic plasma exchange (PLEX). Serum ferritin level drastically decreased by 64% (from 1,109 to 399 ng/ml) after PLEX (*Segment C* in Figure 1). The patient showed no clinical deterioration for the next 4 months, and his respiratory distress slowly improved. However, prior neurological disability including blindness of the right eye, paraplegia, and bladder dysfunction failed to improve.

## Discussion

This case showed a rare combination of NMO and overlapping rheumatic diseases associated with anti-MDA5 antibody, which resulted in serious neurological disability. In the previous report of NMO with anti-MDA5-positive DM, the patient had a good outcome after combined immunotherapy with corticosteroids, rituximab, cyclophosphamide, and PLEX (6). Our current patient also appeared to have a favorable course during the first 2 months after the initiation of immunotherapy. However, NMO relapsed frequently within the following 2 months, whereas symptoms or signs of anti-MDA5-associated diseases were rather silent. In addition, the patient experienced an unexpected exacerbation of NMO and ILD after rituximab induction.

Though the pathogenesis of anti-MDA5-positive DM is largely unknown, the involvement of the hyperactivated interferon system has been suggested (7). Serum level of interferon-alpha (IFN-α) was aberrantly elevated in patients with anti-MDA5-positive DM and shown to be correlated with disease activity (7, 8). Although serum IFN-α level was not measured, it was expected to be elevated in this study. Meanwhile, growing evidence indicates that the elevation of serum IFN-α level might also take part in the pathogenesis or disease activity of anti-AQP4-positive NMO (9–11). There are cases of NMO induced by the therapeutic use of recombinant IFN-α for other diseases (10, 11). Moreover, there was a case of anti-AQP4-positive NMO in a child with increased endogenous IFN-α level (12). Taken together, elevation of IFN-α in the presence of anti-MDA5 antibody might also affect the pathogenesis or high disease activity of NMO in this study.

Serum ferritin is an acute phase reactant induced by various cytokines in inflammatory responses (13, 14). In our case, hyperferritinemia persisted throughout the course of illness without the occurrence of RP-ILD. Moreover, serum ferritin level kept increasing even in the period of symptomatic improvement of ILD, which is not consistent with the fact that serum ferritin is a useful marker for evaluating the therapeutic response of ILD in patients with anti-MDA5-positive DM (4, 15). This discrepancy meant that hyperferritinemia just indicates insufficient control of overall inflammatory conditions, not specifically ILD. Rather, serum ferritin level remained high during the period of frequent relapses or exacerbation of NMO, and it was not until the patient underwent PLEX that serum ferritin level dramatically decreased and there was no more relapse of NMO. Hyperferritinemia and its persistence appeared to be associated with the highly active state of NMO in our patient, though the paucity of the case of NMO with anti-MDA5-positive DM and lack of previous literature made it difficult to determine the relevance between them. Further data on ferritin level in NMO with or without anti-MDA5 antibody are needed.

Rituximab is well-known to be effective in reducing the frequency of relapse and severity in patients with NMO (16, 17). ILD complicated with anti-MDA5-positive DM is often unresponsive to conventional corticosteroid therapy (18). Rituximab might be a promising therapeutic option, as several cases showing its efficacy against refractory ILD with anti-MDA5 antibody have been reported (18–20). However, in our case, the patient experienced unexpected exacerbation of both NMO and ILD in a more severe manner after induction treatment with rituximab. It may just be a natural worsening of both unstable diseases regardless of rituximab infusion. However, the concurrent flare-up of his autoimmune diseases might be related to rituximab induction, given paradoxical exacerbation of NMO, called post-rituximab relapse, has been often reported (21). The presumed mechanism of the phenomenon is associated with B-cell activating factor (BAFF), a crucial regulator of B-cell maturation and antibody production (21, 22). Rituximab can lead to the transient elevation of BAFF within the induction period with a lack of therapeutic efficacy, which is followed by the elevation of anti-AQP4 antibody (22). That is, elevated serum BAFF may stimulate the already existing plasma cells to produce antibodies, thereby contributing to rebound of disease activity (21). This hypothesis is consistent with the finding that the fluorescence intensity of anti-AQP4 antibody was stronger than that measured before rituximab injection in our case. In terms of ILD in our case, what caused the unexpected worsening of ILD after rituximab injection is unclear, with the rarity of similar cases. It is assumed that the post-rituximab elevation of serum BAFF level may be responsible for the exacerbation of ILD, as in post-rituximab NMO relapse, given that BAFF was recently suggested to play a major role in the development of ILD in patients with anti-MDA5-positive DM (7, 23).

In summary, NMO can overlap with anti-MDA5-positive DM and ILD, and its relapse or exacerbation may occur with high disease activity in the presence of anti-MDA5 antibody or hyperferritinemia. In addition, it is worthy to consider that rituximab may contribute to the transient worsening of both NMO and ILD during the early post-induction period.

## Data Availability Statement

The original contributions presented in the study are included in the article/supplementary material, further inquiries can be directed to the corresponding author.

## Ethics Statement

The study was approved by the Institutional Review Board at Chonnam National University Hospital (CNUH-EXP-2020-005). The patient provided written informed consent for the publication of the case report.

## Author Contributions

T-SN: conceptualization and supervision. Y-RK, K-HK, K-HL, KK, and GC: data curation. Y-RK, K-HK, S-JL, and S-YC: formal analysis. Y-RK and T-SN: investigation and visualization. Y-RK, K-HK, and T-SN: writing—original draft. All authors contributed to the article and approved the submitted version.

## Conflict of Interest

The authors declare that the research was conducted in the absence of any commercial or financial relationships that could be construed as a potential conflict of interest.

## References

[1] Nakashima R Imura Y Kobayashi S Yukawa N Yoshifuji H Nojima T. The RIG-I-like receptor IFIH1/MDA5 is a dermatomyositis-specific autoantigen identified by the anti-CADM-140 antibody. Rheumatology. (2010) 49:433–40. 10.1093/rheumatology/kep375 20015976

[2] Sato S Kuwana M Fujita T Suzuki Y. Anti-CADM-140/MDA5 autoantibody titer correlates with disease activity and predicts disease outcome in patients with dermatomyositis and rapidly progressive interstitial lung disease. Mod Rheumatol. (2013) 23:496–502. 10.3109/s10165-012-0663-4 22644102

[3] Li L Wang Q Yang F Wu C Chen S Wen X. Anti-MDA5 antibody as a potential diagnostic and prognostic biomarker in patients with dermatomyositis. Oncotarget. (2017) 8:26552–64. 10.18632/oncotarget.15716 28460448 PMC5432278

[4] Gono T Sato S Kawaguchi Y Kuwana M Hanaoka M Katsumata Y. Anti-MDA5 antibody, ferritin and IL-18 are useful for the evaluation of response to treatment in interstitial lung disease with anti-MDA5 antibody-positive dermatomyositis. Rheumatology. (2012) 51:1563–70. 10.1093/rheumatology/kes102 22589330

[5] Papadopoulos MC Verkman AS Aquaporin 4 and neuromyelitis optica. Lancet Neurol. (2012) 11:535–44. 10.1016/S1474-4422(12)70133-3 22608667 PMC3678971

[6] Delman D Peng X Zedek DC Jewells V Chahin N Markovic-Plese S. Dermatomyositis as a presentation of neuromyelitis optica spectrum disorder. J Neuroimmunol. (2015) 278:108–11. 10.1016/j.jneuroim.2014.07.016 25595259

[7] Zhang SH Zhao Y Xie QB Jiang Y Wu YK Yan B. Aberrant activation of the type I interferon system may contribute to the pathogenesis of anti-melanoma differentiation-associated gene 5 dermatomyositis. Br J Dermatol. (2019) 180:1090–8. 10.1111/bjd.16917 29947075

[8] Horai Y Koga T Fujikawa K Takatani A Nishino A Nakashima Y. Serum interferon-alpha is a useful biomarker in patients with anti-melanoma differentiation-associated gene 5 (MDA5) antibody-positive dermatomyositis. Mod Rheumatol. (2015) 25:85–9. 10.3109/14397595.2014.900843 24716595

[9] Asgari N Voss A Steenstrup T Kyvik KO Stenager E Lillevang ST. Interferon alpha association with neuromyelitis optica. Clin Dev Immunol. (2013) 2013:713519. 10.1155/2013/713519 24348680 PMC3855997

[10] Usmani N McCarthy M Rammohan KW Ortega MR. Fulminant myelitis with NMO IgG antibody following treatment with interferon alpha. J Neurol. (2014) 261:240–1. 10.1007/s00415-013-7202-x 24337354

[11] Williams J McGlasson S Irani S Duffy D Crow Y Hunt D. Neuromyelitis optica in patients with increased interferon alpha concentrations. Lancet Neurol. (2020) 19:31–3. 10.1016/S1474-4422(19)30445-4 31839246

[12] McLellan KE Martin N Davidson JE Cordeiro N Oates BD Neven B. JAK 1/2 blockade in MDA5 gain-of-function. J Clin Immunol. (2018) 38:844–6. 10.1007/s10875-018-0563-2 30443754

[13] Gabay C Kushner I. Acute-phase proteins and other systemic responses to inflammation. N Engl J Med. (1999) 340:448–54. 10.1056/NEJM199902113400607 9971870

[14] Kernan KF Carcillo JA. Hyperferritinemia and inflammation. Int Immunol. (2017) 29:401–9. 10.1093/intimm/dxx031 28541437 PMC5890889

[15] Gono T Kawaguchi Y Satoh T Kuwana M Katsumata Y Takagi K. Clinical manifestation and prognostic factor in anti-melanoma differentiation-associated gene 5 antibody-associated interstitial lung disease as a complication of dermatomyositis. Rheumatology. (2010) 49:1713–9. 10.1093/rheumatology/keq149 20498012

[16] Kim SH Huh SY Lee SJ Joung A Kim HJ. A 5-year follow-up of rituximab treatment in patients with neuromyelitis optica spectrum disorder. JAMA Neurol. (2013) 70:1110–7. 10.1001/jamaneurol.2013.3071 23897062

[17] Gao F Chai B Gu C Wu R Dong T Yao Y. Effectiveness of rituximab in neuromyelitis optica: a meta-analysis. BMC Neurol. (2019) 19:36. 10.1186/s12883-019-1261-2 30841862 PMC6402122

[18] Kawasumi H Gono T Kawaguchi Y Yamanaka H. Recent treatment of interstitial lung disease with idiopathic inflammatory myopathies. Clin Med Insights Circ Respir Pulm Med. (2015) 9(Suppl. 1):9–17. 10.4137/CCRPM.S23313 26279636 PMC4514184

[19] So H Wong VTL Lao VWN Pang HT Yip RML. Rituximab for refractory rapidly progressive interstitial lung disease related to anti-MDA5 antibody-positive amyopathic dermatomyositis. Clin Rheumatol. (2018) 37:1983–9. 10.1007/s10067-018-4122-2 29713969

[20] Tokunaga K Hagino N. Dermatomyositis with rapidly progressive interstitial lung disease treated with rituximab: a report of 3 cases in Japan. Intern Med. (2017) 56:1399–403. 10.2169/internalmedicine.56.7956 28566605 PMC5498206

[21] Perumal JS Kister I Howard J Herbert J. Disease exacerbation after rituximab induction in neuromyelitis optica. Neurol Neuroimmunol Neuroinflamm. (2015) 2:e61. 10.1212/NXI.0000000000000061 25738163 PMC4335814

[22] Nakashima I Takahashi T Cree BA Kim HJ Suzuki C Genain CP. Transient increases in anti-aquaporin-4 antibody titers following rituximab treatment in neuromyelitis optica, in association with elevated serum BAFF levels. J Clin Neurosci. (2011) 18:997–8. 10.1016/j.jocn.2010.12.011 21565508

[23] Matsushita T Kobayashi T Kano M Hamaguchi Y Takehara K. Elevated serum B-cell activating factor levels in patients with dermatomyositis: association with interstitial lung disease. J Dermatol. (2019) 46:1190–6. 10.1111/1346-8138.15117 31631384

